# The effect of training using an upper limb rehabilitation robot (HEXO-UR30A) in chronic stroke patients: A randomized controlled trial

**DOI:** 10.1097/MD.0000000000033246

**Published:** 2023-03-24

**Authors:** Ji Ae Kim, Min Ho Chun, Anna Lee, Younghoon Ji, Hyeyoun Jang, Changsoo Han

**Affiliations:** a Department of Rehabilitation Medicine, Asan Medical Center, University of Ulsan College of Medicine, Seoul, Republic of Korea; b Asan Institute for Life Sciences, Asan Medical Center, Seoul, Republic of Korea; c HEXAR Humancare Co. Ltd, Ansan-si, Korea.

**Keywords:** neurological rehabilitation, robotics, stroke, upper limb

## Abstract

Upper limb robotic rehabilitation can be beneficial to the patients when applied appropriately. HEXO-UR30A is a novel exoskeletal type upper limb rehabilitation robot that provides continuous passive motion to the shoulder joint.

**Objective:**

The purpose of this study is to evaluate the effectiveness of HEXO-UR30A on the patient’s functional change, spasticity, and range of motion (ROM).

**Methods:**

We included stroke patients with upper limb hemiparesis of age > 19 years with spasticity grading of modified Ashworth scale < 3 and Brunnstrom recovery stage ≥ 4. The efficacy of the robot was investigated based on a rehabilitation program for 3 weeks. Patient’s functions were compared before vs after treatment and between the HEXO group vs control. We conducted the Fugl–Meyer Assessment of the Upper Extremity, modified Barthel index, modified Ashworth scale, ROM, and Motricity Index upper limb. Patients’ satisfaction was evaluated using a questionnaire after every 10 sessions of training.

**Results:**

In the HEXO group, the Fugl–Meyer assessment for shoulder improved significantly (*P* value = .006*) compared with the control group (*P* value = .075). Both groups showed significant improvement (*P* value < .05) in Motricity Index upper limb after treatment. There were some improvements in the passive and active ROM. Patients in the HEXO group reported high satisfaction with upper limb rehabilitation.

**Conclusion:**

These results show that HEXO-UR30A can improve functional ability in chronic stroke patients. Moreover, the high satisfaction in patients might promote active involvement in upper limb rehabilitation.

## 1. Introduction

Stroke is a disabling and deleterious disease with high mortality. The survivors often experience affected limb motor impairment, cognitive decline, and other functional disabilities. The global burden of stroke is tremendous.^[1]^ Conventional stroke rehabilitation for the upper limb includes occupational therapy, constraint-induced movement therapy, and electrostimulation therapy.^[2,3]^ The application of the upper limb rehabilitation training robot in stroke patients has been gaining popularity based on its effectiveness in the recovery of motor function as confirmed by previous studies.^[4]^ Robot-assisted rehabilitation can provide better task-oriented, repetitive, and high-intensity training compared to that seen in conventional therapist-assisted training.^[5]^

Rehabilitation robot types include end-effector type and exoskeletal type. Previous studies showed that therapeutic rehabilitation with an exoskeletal robot improved the patient’s upper limb motor function and daily activity function.^[6,7]^ A systematic review with meta-analysis concluded that exoskeletal robots had significantly favorable outcomes on post-stroke upper arm motor function compared to end-effector robots.^[5]^ Veerbeek et al^[8]^ reported that shoulder/elbow robotics improved motor function and muscle strength significantly in stroke patients. On the other hand, elbow/wrist robotics improved only motor control. These findings suggest that exoskeletal shoulder robotics training would help stroke patients’ motor recovery.

Hexo-UR30A is a new exoskeletal robot designed for shoulder joint exercise. It provides continuous isokinetic, isometric, and isotonic movement for the shoulder joint so that patients can move their shoulders at a constant speed without pain. The effectiveness of Hexo-UR30A is yet to be investigated clinically, especially in chronic stroke patients.

The purpose of this study was to quantitatively compare the recovery of shoulder joint range of motion and functional and motor improvement of chronic stroke patients in the conventional upper limb ergometer rehabilitation group and Hexo-UR30A-assisted rehabilitation group.

## 2. Methods

The study design was a single-center, prospective, non-blinded, randomized controlled trial (KCT0006808). The study was approved by the Asan Medical Center Institutional Review Board (no. 2021-0643). Written informed consents were given for all participants.

The inclusion criteria were as follows: patients with upper limb hemiparesis after 12 months of the stroke event, age > 19 upper limb spasticity grade lower <3, and Brunnstrom recovery stages of IV, V, and VI. Exclusion criteria were as follows: patients with the severe cognitive decline with lack of communication, upper extremity musculoskeletal problems, combined upper extremity peripheral nervous system disease, badly progressed upper limb contracture, those with comorbidities who cannot perform upper limb rehabilitation, those involved in other studies, and those a high risk of fracture.

Participants recruited were followed up at the rehabilitation department of the outpatient clinic. The randomization sequence was contrived using Excel 2016 (Microsoft, WA) to allocate patients randomly to the HEXO or control group. All participants underwent a 30-minute physical therapy session 10 times in total in 3 weeks. The HEXO group physical therapy included upper limb exercises provided by Hexo-UR30A. Each program included 5 minutes of warm-up, 5 minutes of cool-down in each, and 20 minutes of main training with Hexo-UR30A including shoulder flexion-extension, abduction-adduction, and internal rotation-external rotation exercise. The angular velocity was 10 degrees for 60 seconds, and each degree to shoulder joint moved was optimized to the maximum extent patients can move and do not feel pain or discomfort. In the control group, patients were instructed to perform upper limb range of motion exercise with an ergometer for 30 minutes. The functional and neurologic parameters were evaluated prior to the start of the treatment (baseline) and after the 3-week training session (post-treatment).

The primary evaluation includes upper limb motor function, assessed using Fugl–Meyer Assessment for the upper extremity and Motricity Index for the upper extremity. The Fugl–Meyer Assessment is one of the most recommended motor function assessments in clinical trials for stroke patients’ rehabilitation.^[9]^ The Fugl–Meyer assessment-upper extremity includes the motor domain, and it measures upper limb coordination, movement, and reflex.^[10]^ The score of Fugl–Meyer assessment-upper extremity ranges from 0 to 66 points; 0 signifies complete hemiplegia, and 66 signifies normal upper limb motor function.^[11]^ We conducted sub-analysis using Fugl–Meyer assessment-upper extremity sub-item appreciating only shoulder; scapular elevation, scapular retraction, shoulder abduction, shoulder external rotation, shoulder adduction with internal rotation, 90° shoulder abduction with the elbow extended, and 90° and 180° shoulder flexion with the elbow extended.^[12]^ Motricity Index for upper extremity measures upper arm motor activity, which includes shoulder abduction, elbow flexion, and pinch grip.^[13,14]^ Each sub-score ranges from 0 to 33 points with a total of 100 point-scale.^[15]^

The secondary evaluation includes the Modified Barthel Index, active range of motion, passive range of motion, spasticity measured using the modified Ashworth scale (MAS) grade, and patients’ satisfaction. Modified Barthel Index evaluated the activity of daily living (ADL) of patients, including personal hygiene and feeding, from 0 points (totally dependent) to 100 (independent).^[16,17]^ Active range of motion and passive range of motion was measured using a goniometer. These included shoulder abduction, adduction, flexion, extension, external rotation, and internal rotation in a 180-degree system evaluated using the American Academy of Orthopedic Surgeons guidelines.^[18]^ The standard starting anatomic position, goniometer position, and normal range of motion were applied to evaluate patients’ shoulder range of motion.^[19]^ The increased muscle tone was measured using the MAS.^[20]^ It ranges from 0 to 4 with the scores of 0, 1, 1+, 2, 3, and 4; “0” signifies no increase in muscle tone, and “4” signifies that the affected region is rigid in flexion and extension.^[21]^ The patients’ satisfaction was also evaluated using a questionnaire,^[22]^ which included aspects of satisfaction, efficiency, and effects (Table 1). Patients in the HEXO group provided responses to the questionnaire after 10 sessions of training. Each question was scored from 0 to 5. When patients strongly disagree with the question, the score was 0, and when patients strongly agree with the question, the score was 5.

**Table 1:** Satisfaction questionnaire.

No.	Domain	Item
1	Satisfaction	Are you satisfied with the overall rehabilitation program?
2	Satisfaction	Are you willing to continue to use HEXO-UR30A upper limb robot?
3	Satisfaction	Would you recommend HEXO-UR30A upper limb robot to someone who wants a similar rehabilitation program?
4	Satisfaction	Do you think HEXO-UR30A upper limb robot helps more than other rehabilitation programs?
5	Efficiency	HEXO-UR30A robot helps your upper limb recovery?
6	Effects	Has the HEXO-UR30A robot rehabilitation had a positive change in your psychological state?
7	Effects	Has the HEXO-UR30A robot rehabilitation had a positive change in your concentration on other rehab programs?
8	Effects	Has the HEXO-UR30A robot rehabilitation had a positive change in your concentration on other rehab programs?

An appropriate sample size based on power analysis was not calculated since the study was designed as a pilot study. Statistical analyses were performed using SPSS statistics version 18.0 (SPSS Inc, Chicago, IL). The characteristics of patients were analyzed using Student *t* test and Mann–Whitney *U* test. The functional, motor, and spasticity measurement changes were compared between 2 groups using the Wilcoxon signed-rank test. The improvement from baseline to post-rehabilitation was compared using the Mann–Whitney test. The MAS was converted to numerical scores (0, 1, 2, 3, 4, and 5).^[23]^ The statistical significance was determined by a *P* value of <.05.

## 3. Results

**Figure 1.**
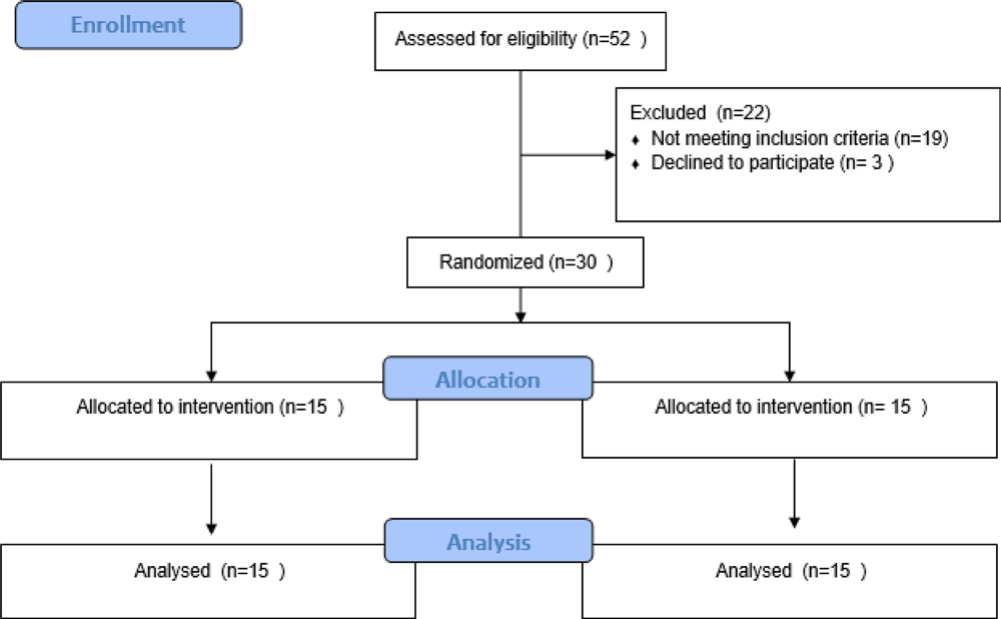
CONSORT flow diagram.

In total, 30 patients were enrolled in the study between June 2021 and September 2021. There was no dropout among patients, and the final analysis included 30 patients (Fig. 1). The age, sex, height, weight, stroke etiology, time post-stroke, and hemiplegia lesion side were not significantly different between groups (Table 2).

**Table 2:** Characteristics of participants.

Variables		HEXO (n = 15)	Control (n = 15)	*P* value
Age (y)		65.53 (8.43)	64.53 (7.72)	.623
Sex (%)	Male	9 (60.0)	9 (60.0)	>.999
	Female	6 (40.0)	6 (40.0)	
Weight (kg)		62.20 (10.22)	69.06 (11.05)	.923
Height (m)		1.60 (0.09)	1.64 (0.09)	.844
Time post-stroke (mo)		214.20 (69.52)	210.60 (80.78)	.399
Stroke etiology, n (%)	Infarction	7 (46.7)	8 (53.3)	.715
	Hemorrhage	8 (53.3)	7 (46.7)	
Hemiplegia lesion side, n (%)	Right	9 (60.0)	8 (53.3)	.713
	Left	6 (40.0)	7 (46.7)	

Values are shown as mean (%).

*P value <.05. For the statistical analysis, the Chi-square test and *t* test were performed.

The primary evaluation, Fugl–Meyer assessment and Motricity index, showed significant improvements after 10 sessions of rehabilitation. However, there were no significant differences between the groups in both evaluations (Table 3). In the HEXO group, the Fugl–Meyer assessment for shoulder improved significantly (*P* value = .006) but not in the control group (*P* value = .075).

**Table 3:** Motor improvement in HEXO and control groups.

	HEXO		Control		
	Baseline	F/U	Baseline	F/U	*P* value
FMA-UE	19.00 [14.00, 24.00]	21.00 [15.00, 27.00]*	23.00 [12.00, 34.00]	23.00 [12.00, 34.00]*	.832
FMA-shoulder	5.00 [4.00, 6.00]	5.00 [4.00, 6.00]*	5.00 [3.00, 7.00]	5.00 [2.00, 8.00]	.539
MI	59.00 [44.00, 74.00]	64.00 [53.00, 75.00]*	58.00 [41.00, 75.00]	59.00 [42.00, 76.00]*	.942

Values are shown as median, and interquartile ranges are in square brackets. FMA-UE, MBI, Modified Barthel Index, MI.

* *P* value < .05 for baseline versus follow-up, the Wilcoxon signed-rank test was performed.

† *P* value < .05 for the difference (follow-up baseline) between the HEXA and control groups, the Mann–Whitney test was performed.

The secondary outcome, the modified Barthel index, was not significantly improved in both groups. In the HEXO group, the active range of motion of shoulder flexion, passive range of motion of shoulder flexion, extension, adduction, and abduction significantly increased after 10 sessions of rehabilitation. Similarly in the control group, all the above active range of motions except shoulder internal rotation significantly improved (Tables 4 and 5). The upper limb spasticity measure in MAS was not significantly different before and after rehabilitation in both groups (Table 6). In the HEXO group, the mean satisfaction, efficiency, and effects scores were 4.85 ± 0.40, 4.80 ± 0.41, and 4.80 ± 0.45, respectively.

**Table 4:** Active range of motion.

	HEXO		Control		
	Baseline	F/U	Baseline	F/U	*P* value
Shoulder flexor	69.00 [29.00, 109.00]	70.00 [40.00, 100.00]*	68.00 [11.00, 125.00]	76.00 [24.00, 128.00]	.512
Shoulder extensor	23.00 [11.00, 37.00]	25.00 [19.00, 31.00]	25.00 [14.00, 36.00]	25.00 [17.00, 33.00]*	.683
Shoulder abductor	59.00 [29.00, 84.00]	56.00 [20.00, 92.00]	55.00 [10.00, 100.00]	60.00 [18.00, 102.00]*	.461
Shoulder adductor	27.00 [18.00, 36.00]	27.00 [19.00, 35.00]	27.00 [15.00, 39.00]	29.00 [18.00, 40.00]*	.806
Shoulder ext. rot	15.00 [4.00, 26.00]	15.00 [-1.00, 31.00]	14.00 [-6.00, 34.00]	18.00 [-3.00, 39.00]*	.187
Shoulder int. rot	27.00 [9.00, 45.00]	30.00 [12.00, 48.00]	16.00 [2.00, 30.00]	16.00 [5.00, 27.00]	.061

Values are shown as median, and interquartile ranges are in square brackets.

* *P* value < .05 for baseline versus follow-up, the Wilcoxon signed-rank test was performed.

† *P* value < .05 for the difference (follow-up baseline) between the HEXA and control groups, the Mann–Whitney test was performed.

**Table 5:** The difference before and after rehabilitation of a passive range of motion between the HEXO and control groups.

	HEXO		Control		
	Baseline	F/U	Baseline	F/U	*P* value
Shoulder flexor	90.00 [63.00, 117.00]	97.00 [72.00, .00]*	96.00 [78.00, 114.00]	104.00 [84.00, 124.00]*	.148
Shoulder extensor	27.00 [11.00, 43.00]	31.00 [23.00, 39.00]*	30.00 [19.00, 41.00]	33.00 [23.00, 43.00]*	.967
Shoulder abductor	77.00 [45.00, 109.00]	84.00 [53.00, 115.00]*	67.00 [31.00, 103.00]	89.00 [53.00, 125.00]*	.775
Shoulder adductor	34.00 [27.00, 41.00]	35.00 [30.00, 40.00]*	32.00 [23.00, 41.00]	35.00 [25.00, 45.00]*	.389
Shoulder ext. rot	35.00 [17.00, 53.00]	39.00 [20.00, 58.00]	38.00 [14.00, 62.00]	42.00 [18.00, 66.00]*	.935
Shoulder int. rot	27.00 [9.00, 45.00]	30.00 [12.00, 48.00]	16.00 [2.00, 30.00]	16.00 [5.00, 27.00]	.061

Values are shown as median, and interquartile ranges are in square brackets.

* *P* value < .05 for baseline versus follow-up, the Wilcoxon signed-rank test was performed.

† *P* value < .05 for the difference (follow-up baseline) between the HEXO and control groups, the Mann–Whitney test was performed.

**Table 6:** Spasticity improvement before and after the rehabilitation training between the HEXO and control groups.

	HEXO			Control		
	Baseline	F/U	*P* value	Baseline	F/U	*P* value
Shoulder flexor	1.00 [0.00, 2.00]	1.00 [0.00, 2.00]	.564	2.00 [1.00, 3.00]	2.00 [1.00, 3.00]	.317
Shoulder extensor	1.00 [0.00, 2.00]	1.00 [0.00, 2.00]	.564	2.00 [0.00, 4.00]	2.00 [1.00, 3.00]	.564
Shoulder abductor	2.00 [2.00, 2.00]	2.00 [1.00, 3.00]	.083	2.00 [2.00, 2.00]	2.00 [1.00, 3.00]	.157
Shoulder adductor	1.00 [0.00, 2.00]	1.00 [0.00, 2.00]	.317	2.00 [1.00, 3.00]	2.00 [1.00, 3.00]	.317
Shoulder ext. rot	2.00 [1.00, 3.00]	2.00 [2.00, 2.00]	.317	2.00 [1.00, 3.00]	2.00 [0.00, 4.00]	.564
Shoulder int. rot	1.00 [0.00, 2.00]	1.00 [0.00, 2.00]	.317	2.00 [2.00, 2.00]	2.00 [2.00, 2.00]	.317

Values are shown as median, and interquartile ranges are in square brackets. For the statistical analyses, the Wilcoxon signed-rank test was performed.

## 4. Discussion

The present study compared exoskeletal robot-assisted upper limb rehabilitation with conventional upper limb ergometer physiotherapy and demonstrated that HEXO-UR30A was also effective for chronic stroke patients. The upper limb motor function of patients improved in both groups; however, the function of active daily living did not improve in both groups.

In a previous study, Lynch et al^[24]^ discovered that the device provided continuous passive motion rehabilitation and improved shoulder joint instability but showed no significant improvement in motor function, spasticity, and self-care function. Our study findings support this in that the spasticity and self-care function score did not improve after robot-assisted continuous passive movement exercises of the shoulder. In contrast, motor function improved significantly in this study as demonstrated by the Fugl–Meyer assessment and Motricity Index. The HEXO-UR30A provided more sophisticated continuous movement exercises of the shoulder, namely flexion-extension, abduction-adduction, and external rotation-internal rotation, compared to previous research, which used a continuous passive motion machine that only provided 90-degree flexion and 80-degree external rotation.

Our findings demonstrated that the motor function of chronic patients who received physiotherapy with exoskeletal robot-assisted continuous passive movement significantly improved. Lindberg et al^[25]^ provided 20 minutes of passive movement exercises and 5 minutes of assisted active movements and suggested that task-specific rehabilitation using a robot may yield similar results. The upper limb passive and active movement training increased cortical excitability of the sensorimotor and prefrontal areas in the brain, which lead to improvements in the upper limb movement function in chronic stroke patients. This may suggest the underlying mechanism of how the training contributes to the motor function recovery.

HEXO-UR30A training targets specifically the shoulder, and it seems that it improves motor control of the shoulder compared to the conventional ergometer training. Sub-analysis showed shoulder Fugl–Meyer assessment (FMA) improved significantly in the HEXO group but not in the control group. A meta-analysis reported that shoulder/elbow robotics showed significant effects on FMA and FMA-shoulder/elbow/coordination subscale. The difference in FMA-shoulder/elbow/coordination subscale was about 5% in the total score of 2.15 points.^[8]^ Similarly, there was a significant improvement in FMA and FMA shoulder item subscale; however, the difference was small. In both studies, the effect on ADL was not documented. This may be owing to the small motor improvement, which was not sufficient for functional recovery.

The shoulder range of motion improved in both the control and HEXO groups. This could be because HEXO-UR30A sets the range of motion to the point where patients can move painlessly and comfortably, unlike the control group where patients move their shoulders according to the ergometer arm pedal height, which is set at 60 degrees. Although the median passive and active range of motion of shoulder flexion in the control group is higher than 60 degrees, there were patients whose range of motion was less than 60 degrees. Passively stretching the shoulder more than what patients can move might lead to an increase in the shoulder range of motion. Brokaw et al^[26]^ reported that an exoskeletal hand rehabilitation robot that provides a passive range of motion training helped stroke patients increase their range of motion. This study suggests that exoskeletal robots targeting mainly the shoulder can also improve the joint range of motion.

Limitations of this study include a small sample size because of which the statistical power might be low. Future studies with a larger sample size will be warranted. Moreover, the participants who were enrolled in this study were generally good in the function of daily activity where the median modified Barthel index was over 90. This may explain why there was no significant improvement in ADL. There is a plateau in function recovery, and better function at a high-demanding skill is more difficult to achieve. Finally, the investigator who evaluated the patients was not blinded to patients group allocation, and this might affect the measurement. Further studies where the investigator is blinded to group allocation are warranted.

In conclusion, the novel HEXO-UR30A can be effective in the improvement of shoulder motor functions in chronic stroke patients. The daily activity function, spasticity, and range of motion did not improve in both the HEXO and control groups. Patients who received HEXO-UR30A assisted rehabilitation reported high satisfaction, which might improve patient involvement in upper limb rehabilitation.

## Acknowledgments

The authors would like to thank Younghoon Ji, Changsoo Han, Hyeyoun Jang for their technical assistance and Ahro Lee, Anna Lee with the study interventions included in this investigation. J.A.K. wrote the paper itself, and M.H.C. is the guarantor. All of the authors initiated the study, designed it, and monitored progress together.

## Author contributions

**Conceptualization:** Ji Ae Kim, Min Ho Chun.

**Data curation:** Ji Ae Kim, Min Ho Chun.

**Formal analysis:** Min Ho Chun.

**Funding acquisition:** Min Ho Chun.

**Methodology:** Anna Lee.

**Project administration:** Anna Lee, Changsoo Han.

**Resources:** Younghoon Ji, Hyeyoun Jang, Changsoo Han.

**Software:** Anna Lee, Younghoon Ji, Hyeyoun Jang, Changsoo Han.

**Supervision:** Younghoon Ji, Hyeyoun Jang.

**Validation:** Hyeyoun Jang.

**Writing – original draft:** Ji Ae Kim.

**Writing – review & editing:** Ji Ae Kim.
